# CAR-T Cells in Canada; Perspective on How to Ensure We Get Our Value’s Worth

**DOI:** 10.3390/curroncol30040305

**Published:** 2023-04-03

**Authors:** Pierre J. A. Villeneuve, Christopher Bredeson

**Affiliations:** 1 Division of Hematology, The Ottawa Hospital, Ottawa, ON K1H 8L6, Canada; 2 School of Epidemiology and Public Health, University of Ottawa, Ottawa, ON K1G 5Z3, Canada

**Keywords:** CAR-T, CADTH, health technology assessment, HTA

## Abstract

New therapies in a publicly funded healthcare system are first appraised by health technology assessment agencies that provide funding recommendations to the payers. Treatment with Chimeric Antigen Receptor-T cell (CAR-T) therapy is revolutionizing the management of patients with relapsed/refractory aggressive B-cell lymphoma by providing an effective alternative to the standard of care. Yet, the implementation of CAR-T treatment has a substantial impact on the healthcare system due to its high cost, complex manufacturing process, and requirement for highly specialized services and expertise. CAR-T Cells, as a “living drug”, are fundamentally different from usual medications, and their approvals and funding recommendations pose unique challenges to the health technology agency. In this paper, we explore the specific challenges that face the health technology agencies in reviewing reimbursement recommendations for CAR-T therapy. We take a Canadian perspective and use CAR-T treatment of relapse/refractory aggressive B-cell lymphoma as an example.

## 1. Introduction

Decades of research to harness the potential of the immune system have culminated in the development of Chimeric Antigen Receptor T cell (CAR-T) therapy, which is now revolutionizing the management of patients with malignancies, most notably aggressive B-cell lymphoma and acute lymphoblastic leukemia. CAR-T cells provide patients who have exhausted curative-intent chemotherapy options and chance for long-term survival [1,2].

CAR-T cells are game-changers, but their implementation and availability come with significant challenges due, in part, to high costs and the complexity associated with CAR-T cell manufacturing and treatment that result in broader, adverse impacts on the healthcare system. The number of indications for CAR-T within malignant hematology—and more broadly within oncology—will grow rapidly over the next decade; a recent search with Clinicaltrials.gov (accessed on 12 February 2023) showed that there are well over 1000 trials currently registered. These include CAR-T trials for the treatment of both hematological and solid malignancies that encompass a wide array of additional strategies, using other tumor-associated antigens than CD19 and in combination with additional therapies, including tyrosine kinase inhibitors and checkpoint inhibitors in the case of hepatocellular carcinoma [3,4]. It is an opportune time to review the challenges and potential for widespread and efficient use of CAR-T in an equitable and sustainable fashion. There are important questions that need to be answered to ensure we develop equitable access and sustainable use of CAR-T in a public payer healthcare system such as Canada. This will ensure that the expansion of public funding of CAR-T cell programs is done with the consideration of value for money while also improving the overall health of individuals living with malignancies. The case of CD19-directed CAR-T cells for the management of relapse/refractory (r/r) diffuse large B-cell lymphoma (DLBCL) from a Canadian perspective will be used as a demonstration case.

## 2. Manufacturing and Delivery of CAR-T Cells

CAR-T cell manufacturing involves a complex series of coordinated and highly specialized steps and expertise. The manufacture of the currently approved commercial CAR-T cell products consists of using genetically engineered autologous lymphocytes that have been modified to harbor a T-cell receptor that recognizes a specific antigen, most commonly the CD19 antigen—a key antigen expressed on DLBCL and B-cell acute lymphoblastic leukemia (B-ALL). CAR-T cells are manufactured from autologous lymphocytes collected through leukapheresis of the patient, transduced ex vivo via a clinical-grade virus with an anti-CD19 T-cell receptor and expanded in a GMP-grade facility before they are ready to be infused back to the patient. Prior to the infusion of CAR-T cells, patients undergo lymphodepletion with chemotherapy, most commonly cyclophosphamide and fludarabine [2,5]. This process can take days to weeks, depending on the manufacturing platform that is utilized. While patients wait for their CAR-T cells to be manufactured, many will undergo bridging chemotherapy and/or radiotherapy to keep their disease under control until the CAR-T cell infusion. The percentage of patients receiving bridging treatments is variable from study to study and is not consistently reported but it has been as high as 90% [6]. In clinical trials, the proportion of patients who had their leukocytes collected but did not receive their CAR-T cells because of progressive disease is also variable [1,2].

## 3. Clinical Effectiveness of CAR-T Cells

The evidence demonstrating clinical effectiveness of CAR-T cells that led to Health Canada approval for the management of r/r DLBCL was derived from single-arm phase 1–2 studies that showed response rates above 50% [7]. The best attempt to compare the relative effectiveness of CAR-T cells against standard of care (SOC) stems from indirect comparisons. The most notable comparator to CAR-T therapy has been the historical control of patient outcomes reported in the SCHOLAR study, an international, multicentre retrospective, pooled analysis of patients with DLBCL who either had primary refractory disease or who relapsed within 12 months of standard chemotherapy [8,9]. The SCHOLAR patient population differs in some respects to the one that receive publicly funded CAR-T cells in Canada for the treatment of relapse/refractory DLBCL since it excluded patients who relapsed after 12 months, thus affecting the validity of the comparison.

The first two CAR-T cells approved and commercially available in Canada were tisagenlecleucel (KymriahTM, Novartis, Morris Plains, NJ, USA) and axicabtagene ciloleucel (YescartaTM, Gilead Sciences Canada, El Segundo, CA, USA), approved by Health Canada in 2018 and 2019, respectively. Both are second-generation anti-CD19 CAR-T cells [1,2], approved for adult patients with r/r DLBCL, primary mediastinal B-cell lymphoma and transformed follicular lymphoma. Estimates of the incremental expenditure associated with the reimbursement of tisagenlecleucel and axicabtagene ciloleucel alone in adult patients with r/r DLBCL in Canada is in the order of 840 million Canadian dollars for the first three years [10,11]. This figure likely underestimates longer-term projections, as clinical indications for CAR-T cell therapy continue to rapidly expand to include other targeted malignancies. Also, mounting evidence suggests that dose-escalation of CAR-T may be associated with improved outcome, potentially at the expense of further increasing therapy-related costs [12,13]. In the context of finite healthcare resources, the sustainability of a publicly funded program relies on ensuring an equitable review of how funding allocation for CAR-T cells is being performed.

## 4. Drug Review Process in Canada

To understand how decisions are made for the reimbursement of new drugs in Canada, it is important to review the process through which new pharmaceutical agents are appraised to inform funding recommendations in Canada. New drugs approved by Health Canada are submitted by the manufacturer to the Canadian Agency for Drugs and Technology (CADTH), an arms-length, federally funded agency mandated to independently review new drugs and provide funding recommendations for public reimbursement in all provinces and territories, except the province of Quebec. The CADTH review process is a thorough and systematic process that relies on a deliberative framework that is systematically applied to compare the submitted drug to the relevant standards of care (SOC). There are four axes to this framework:

(1) Clinical evidence: The comparative effectiveness of the new, submitted drug is compared to the SOC by a group of clinical experts and methodologists based on available evidence. Although overall survival has been the preferred outcome to inform recommendations, relevant surrogate outcomes, including health-related quality of life (HRQOL), toxicity, and disease-free survival, are commonly relied upon to allow comparison of effectiveness. The comparative effectiveness of the submitted drug compared to the SOC is performed. Standard, evidence-based criteria are used to assess the quality of the evidence in terms of their validity as they apply to the Canadian patient population, uncertainty around estimates, appropriateness of comparators, and clinical relevance of the outcomes being considered, with thorough documentation of this review process in the final, public-facing report.
(2) Economic evaluation: The economic impact of the drug under review is compared to the SOC via a cost-effectiveness analysis (CEA). The CEA relies on a model prepared by the submitter and critically reviewed by CADTH’s economic experts. The assumptions made in the model are reviewed with clinical experts, and sensitivity analyses are performed by the committee’s methodologists to assess the impact of key variables, including drug prices, estimates of clinical benefits used to populate the model, time horizon, and overall uncertainty of the CEA. It is important to point out that the objective of the economic analysis, by virtue of its design, is to provide a good estimate of the economic value of the submitted drug for the specific indication(s) compared to the SOC; the CEA is not designed to inform on the economic value compared to other therapeutic options and/or different indications.
(3) Adoption feasibility: This third axis evaluated by CADTH considers factors that would impact the feasibility and availability of the new treatment option. These include mode of drug administration, availability of appropriate expertise at local, regional, and/or provincial/territorial levels, global budget impacts, and additional investigations for drug initiation, monitoring, and/or related complications in addition to those required with SOC. This consideration is in part to ensure national-level equity in who can access the proposed new treatment as well as planning for resources as appropriate for the provinces.
(4) Patient values: Patient values are assessed through a questionnaire through which patients and/or patient advocacy partners are given an opportunity to provide written feedback on the value of the submitted therapy from their perspective. However, patient values are difficult to quantify, which makes it challenging when wanting to compare different treatments, and this aspect may limit how patient values inform reimbursement decisions.

An interdisciplinary expert committee weighs in on all four criteria and deliberates to provide a formal recommendation to CADTH: reimburse, with no conditions; do not fund; conditional recommendation, pending price negotiations; and/or review of additional clinical evidence. These recommendations are published and made available to stakeholders and are enforced by the provinces and territories most of the time [14].

The current CADTH appraisal framework has proven effective at informing the provinces of appropriate funding recommendations over the last decade [14]. To appraise the value-added of CAR-T cells among publicly funded therapies for targeted malignancies and clinical indications, there is a need for longer-term data on the economic and clinical impact of CAR-T cells. The framework for drug review needs to adhere to all four axes as well as consider opportunity costs or the costs of therapies forgone to be able to fund CAR-T cell therapies.

## 5. Uncertainty around Clinical Benefit

The value of the economic and clinical assessment hinge on the quality of the available clinical data; the greater the degree of certainty around the clinical outcomes, the more accurate the estimate of treatment benefit. In the case of CAR-T cells, there remains uncertainty about the extent of the benefits. At the time of submission to the Canadian HTA, the estimated effectiveness of CAR-T cells in the management of r/r aggressive B-cell lymphoma was derived from single-arm studies. The comparative effectiveness of CAR-T cells with SOC for the treatment of r/r DLBCL was derived from an indirect matched comparison to the outcome of patients with aggressive B-cell lymphoma enrolled in the SCHOLAR study (described above) [8,9]. The SCHOLAR study’s patient population included patients with arguably higher-risk disease compared to those who received publicly funded CAR-T cells in Canada. The discrepancy between the two patient populations potentially biases the estimate of the relative benefit in favour of CAR-T cells compared to SOC. A practical alternative approach could be to compare outcomes among patients treated with CAR-T cells against historical controls, matched by demographics and disease characteristics using propensity score matching. This approach requires access to comprehensive databases containing information on patient demographics and disease characteristics. These variables could be supplemented by linking them to patients’ outcomes and toxicities. The potential biases due to inherent differences in patients’ demographics, comorbidities, and disease characteristics between the two populations could be accounted for by performing an indirect match comparison using a propensity score. Recently published randomized studies comparing tisagenlecleucel (BELINDA trial) and axicabtagene ciloleucel (ZUMA-7 trial) to SOC provide helpful information on the clinical effectiveness of CAR-T therapy; interestingly, the two studies resulted in seemingly contradictory results; In the BELINDA trial, where tisagenlecleucel was compared head-to-head with SOC, CAR-T therapy was not superior compared to SOC [15]. This is probably due, at least in part, to the fact that patients enrolled in the BELINDA trial have higher-risk features than those enrolled in the ZUMA-7 trial, thus possibly skewing the results unfavorably for CAR-T therapy [16]. The ZUMA-7 study did demonstrate superior clinical effectiveness compared to SOC, although that was not the case in patients who had bulky or rapidly progressing disease [17].

The degree of uncertainty around the clinical and economic impact of CAR-T cells is even greater in the longer term since clinical data to date extend to only 5 years [18]. The economic models that CADTH relies on for the extrapolation of the long-term impact of anti-CD19 CAR-T cells essentially assume that the initial remission rates seen within the first few years are preserved over an extended time horizon of 20 years [10,11]. The results of these economic models are sensitive to the duration of the time horizon along with the survival and progression-free survival rates [10,11]. Given the absence of data on the long-term benefit and toxicity profile of CAR-T cells, it is crucial that patients treated with CAR-T cells within the context of clinical trials continue to be followed to obtain a more accurate estimate of the evolution of therapeutic benefits and adverse events over time. These longer-term clinical findings and associated costs can inform funding recommendations to ensure good value for money for CAR-T cell therapy.

CAR-T cell-treated patients report dramatic changes in HRQOL related to the cumulative benefit of CAR-T cells on disease control and of the short and long-term toxicities from the cellular treatment [19,20]. The estimate of HRQOL can be translated into a utility score to reflect the level of physical, mental, and social functioning associated with a particular health state and be incorporated into the economic evaluation. So far, most studies have demonstrated improvement in HRQOL with CAR-T cell treatment in patients with r/r DLBCL, but the results published so far lack validity for patients who receive publicly funded CAR-T therapy in Canada, since only patients with aggressive B-cell lymphoma who are either primary refractory or who have relapsed after two lines of therapy are eligible for CAR-T reimbursement. [19,21,22,23,24,25].

In summary, the uncertainty around the clinical and economic impact of CAR-T cell therapy can be addressed with longer-term follow-up of patients enrolled in clinical trials as well as the collection of real-world evidence prospectively with propensity score-matched analyses. This approach would ensure we derive a better estimate of the clinical impact of CAR-T cells, both in terms of benefits and adverse events, with a population of enrolled patients that reflects what is publicly funded. This approach could also provide an opportunity to compare the outcomes of these CAR-T cell-treated patients with historical control from patients treated from the same institutions and who harbor the same disease characteristics. The outcome of these historical control patients could be compared using a matched propensity score to those treated with CAR-T cells, thus offering a better estimate of the clinical impact of CAR-T cell treatments. Moreover, a real-world evidence study would provide an important opportunity to examine societal costs associated with CAR-T cell therapy, including patient and caregiver data on out-of-pocket costs, resource utilization outside of the healthcare setting, and loss of productivity.

## 6. Review Framework to Provide Funding Recommendations

The current CADTH framework, as well as that of other Health Technology Assessment (HTA) agencies not discussed here, is, by design, best suited to inform the relative value of a new treatment compared to an SOC. This approach is appropriate in most instances of submissions for new drugs when the proposed new treatment and the SOC being compared share similarities in clinical impact (benefits and adverse events) and economic value. It can be argued that the CADTH framework may not be fully suited for highly disruptive treatments such as CAR-T cells, as the reimbursement of CAR-T cells will have an unprecedented impact on patient outcome, overall healthcare budget, and healthcare resource utilization, with downstream consequences on public funding of new beneficial therapies for malignancies and, more broadly, other health conditions. A significant additional limitation of CADTH’s framework lies in the fact that the patient values assessment relies on feedback from patients or patient advocacy groups that may not be standardized and comparable across clinical conditions or economic evaluations. Although patients’ value is, in principle, given equal weight to the other arms of the framework, they may, in some circumstances, lack the necessary discriminative properties to be as informative as they should be in decision-making.

The choice of an ideal decision framework for an HTA is complex and characterized by a series of trade-offs, from the choice of the parameters being considered, their respective weights, and whether the final recommendations result from a deliberative process versus a scoring system. None of the frameworks used in North America or Europe would allow a sufficiently comprehensive, accurate, or informative measure of the opportunity cost of CAR-T cells that would take into consideration not only the clinical benefit of CAR-T cells but also their toxicity, impact on HRQOL, and budget impact to ensure choices that will lead to an improvement for the welfare of the population of interest. From a Canadian perspective, it may be easier to identify the limitations of our current framework and formulate solutions that will improve it.

A deep reform of the HTA framework used by CADTH would prove complex, requiring a lengthy consultative process involving all stakeholders and experts, followed by a thorough process of validation which would take years to complete, whereas the need to inform decisions around the use of CAR-T cells is pressing. Until then, it may be opportune and more practical to focus on improving the patient-value arm by formalizing a patient-value framework that allows patients and patient advocacy groups to provide a more discriminative score to inform the deliberative process. There are investigators conducting a series of standardized interviews on patients with cancer designed to determine what patients value.

## 7. Match Reimbursement with Value for Money

As discussed above, while the short-term benefits of CAR-T cells are clear, the estimate of the long-term benefits and costs of CAR-T cells remain uncertain. Even in the case of the best-case scenarios, a good proportion of patients do not derive long-term benefits from the treatment, with, for instance, up to 20% of patients with r/r DLBCL not demonstrating a clinically significant response [1]. Several risk-sharing agreements have been explored by various jurisdictions to reduce the financial risk associated with the reimbursement of CAR-T cells, including a “pay per performance scheme”, where the CAR-T cell providers are reimbursed only when effectiveness is demonstrated in patients [26]. This risk-sharing strategy is intended to be cost-saving to the payer, but the inefficiency resulting from patients who did not derive benefit after receiving treatment nonetheless does result in costs to the healthcare system, patients, and their families. In addition, it is likely that the costs endured by the CAR-T cell manufacturer have been accounted for in the price negotiation. True cost-saving strategies may in fact depend on prospectively being able to discriminate between patients who are likely to be responders versus those who are not. Additional clinical data derived from clinical trials and prospective real-world evidence may prove informative in identifying these patient subgroups.

## 8. Reduction in Costs of CAR-T Cells

The complex generation of CAR-T cells through centralized, third-party manufacturing has been used as justification for the high costs of this therapy. There is interest in working within academic centres to generate these CAR-T products through point-of-care manufacturing with closed benchtop systems. This has been done in Spain, for example [27,28]. In Canada, the ExCELLirate Canada Platform and the CLIC (Canadian Led Immunotherapies in Cancer) program are also working toward Canadian-made CAR-T products within the academic setting, which will hopefully prove to be cheaper than but equally effective as existing commercial products [29]. This could be a game-changer for the economic impact of CAR-T cells, assuming the incidence and severity related to adverse events with this manufacturing model are unchanged.

## 9. Conclusions

CAR-T cells are a profoundly disruptive technology that offers new therapeutic opportunities but at a very high up-front cost. Although it is clear that CAR-T therapies are game-changers, there remains uncertainty in the estimates of their short- and longer-term clinical and economic benefits and toxicities. There are significant challenges in determining their value to inform the allocation of finite healthcare resources. The evaluation of CAR-T cell therapies provides a unique opportunity to critically evaluate and adapt funding review approaches to ensure patients can access disruptive therapies while preserving our healthcare system.

## Data Availability

Not applicable.
